# Perceived Social Support Protects Lonely People Against COVID-19 Anxiety: A Three-Wave Longitudinal Study in China

**DOI:** 10.3389/fpsyg.2020.566965

**Published:** 2020-11-06

**Authors:** Jianjie Xu, Jingyi Ou, Shuyi Luo, Zhuojun Wang, Edward Chang, Claire Novak, Jingyi Shen, Shaoying Zheng, Yinan Wang

**Affiliations:** ^1^Faculty of Psychology, Beijing Normal University, Beijing, China; ^2^Department of Psychology, University of Michigan, Ann Arbor, MI, United States; ^3^Beijing Key Laboratory of Applied Experimental Psychology, Faculty of Psychology, National Demonstration Center for Experimental Psychology Education, Beijing Normal University, Beijing, China

**Keywords:** social support, loneliness, COVID-19 pandemic, anxiety, longitudinal design

## Abstract

The isolation necessary to prevent the spread of the coronavirus disease 2019 (COVID-19) can give rise to anxiety, especially for lonely people who often feel upset without others’ company. Although isolated from others, people can still receive support from others, which might lower their COVID-19 anxiety. To examine the relationship between loneliness, perceived social support, and anxiety, we measured 222 Chinese participants’ (54.50% female, *M*_*age*_ = 31.53, *SD* = 8.17) trait loneliness, chronic anxiety before the outbreak, COVID-19 anxiety at the peak and decline stages of COVID-19, and their perceived social support across the three time points. The results showed that people’s perceived social support dramatically increased from the pre-pandemic to the peak COVID-19 stage, and remained stable during the decline of COVID-19 stage. In contrast, COVID-19 anxiety decreased from the peak to the decline stage. Further, perceived social support consistently moderated the relationship between loneliness with both chronic anxiety and COVID-19 anxiety. The current study provides initial evidence that perceived social support provides protection for lonely people in daily life as well as during unexpected disasters, which will contribute to finding ways to alleviate lonely people’s anxiety during this global health crisis.

## Introduction

In January 2020, coronavirus disease-2019 (COVID-19) broke out and spread rapidly across the world within 2 months. On March 11th, the World Health Organization (WHO) declared COVID-19 as a pandemic (World Health Organization, 2020c). Up to the end of May 2020, there have been more than 5,560,000 confirmed cases and 351,000 deaths worldwide (World Health Organization, 2020b). As a global health crisis, the COVID-19 epidemic has threatened people’s livelihoods and could give rise to greater anxiety (Bao et al., 2020; Galea et al., 2020).

Anxiety is an emotional state characterized by feelings of tension and apprehension, which reflect the complex emotional reaction under stressful situations (Spielberger et al., 1971). It is a common experience among both COVID-19 patients and the uninfected public (Holmes et al., 2020; Rogers et al., 2020). In China, a representative survey from 7,236 participants of various occupations reported that 35.1% of the sample had at least moderate levels of anxiety symptoms during the outbreak period of COVID-19 (Huang and Zhao, 2020). Likewise, a representative community sample in the United Kingdom reported higher levels of anxiety and trauma symptoms during the COVID-19 period as compared to previous population studies (Shevlin et al., 2020). Similar results were also found in the US, India, and many other countries (Ford et al., 2020; Holmes et al., 2020; Othman, 2020; Roy et al., 2020).

To prevent the pandemic from spreading further, the World Health Organization (2020a) suggested that everyone maintain social distancing and avoid going to crowded places. In China, the government restricted all public transit from January 24th and shut down all non-essential companies and schools (Bureau of Disease Prevention and Control, 2020). In Italy, the government locked down the whole country and enforced a decree to prohibit people from public gathering (Pancani et al., 2020). Overall, billions of people sheltered in place to comply with home quarantine (Banerjee and Rai, 2020; Greenstone and Nigam, 2020). Being confined to the home can lead to deprivation of face-to-face communication, loss of social network size, and lower social contact frequency, which have been linked to increased anxiety (Bao et al., 2020; Brooks et al., 2020; Galea et al., 2020).

Isolation from others is particularly difficult for lonely people, who might often feel upset over the lack of others’ company (Chen et al., 2012). People with high levels of loneliness have a subjective perception of the discrepancy between their desired and actual social relationships (Peplau and Perlman, 1982). According to the Loneliness Model (Hawkley and Cacioppo, 2010), lonely individuals suffer impairments in attention, cognition, behavior, and emotion systems, leading to poor mental health and disorders such as anxiety. Considering that lonely people might possess an excessive desire for social interaction, it is plausible that they might have more anxiety when being isolated from others during the COVID-19 pandemic (Spithoven et al., 2017; Armitage and Nellums, 2020; Holmes et al., 2020).

Though isolation may be challenging for lonely people in particular, they still have other opportunities, aside from face-to-face communication, to perceive other’s support even while being isolated. In general, perceived social support might lower lonely people’s anxiety as a positive psychological resource (Masten, 2001; Taylor and Broffman, 2011; Oh et al., 2014). According to the Salutogenic Model (Antonovsky, 1987), social support is one of the most important general resistance resources, which could prompt people to perceive their lives as predictable, controllable, and understandable, thus performing more adaptively in stressful situations. Similarly, the buffering hypothesis (Cohen and Pressman, 2004) suggests that social support might mitigate the negative effects of risk factors on adjustment. Indeed, recent evidence has shown that social support buffered the detrimental effects of acute stress reaction on COVID-19 anxiety among Chinese people (Guo et al., 2020). Hence, we predicted that social support might moderate the relationship between loneliness and COVID-19 anxiety, as well as chronic anxiety. Furthermore, we want to determine whether people’s perceived social support would fluctuate with different development stages of the COVID-19 pandemic. If so, then we would examine whether the moderating effect of social support in the prediction of trait loneliness to COVID-19 anxiety remains robust across different stages of the COVID-19 pandemic.

The current study design involved the collection of three waves of data at the pre-pandemic, peak, and decline stages of COVID-19 in China to examine the relationship between loneliness, perceived social support, and anxiety. We aimed to examine: (1) whether perceived social support would moderate the relationship between trait loneliness and chronic anxiety in general; (2) whether perceived social support might fluctuate with the different stages of COVID-19 pandemic; and, if yes, and (3) whether perceived social support would moderate the relationship between trait loneliness and COVID-19 anxiety across peak and decline stages of the COVID-19 pandemic.

## Materials and Methods

### Participants and Procedures

The present three-wave data belongs to a longitudinal project concerning the relationship between loneliness, perceived social support, and anxiety. After giving consent, participants filled out the questionnaire through a survey website^1^, and were compensated with 12 yuan (approximately $2) each time. The procedures of the present study were approved by the institutional review board of Beijing Normal University.

**FIGURE 1:**
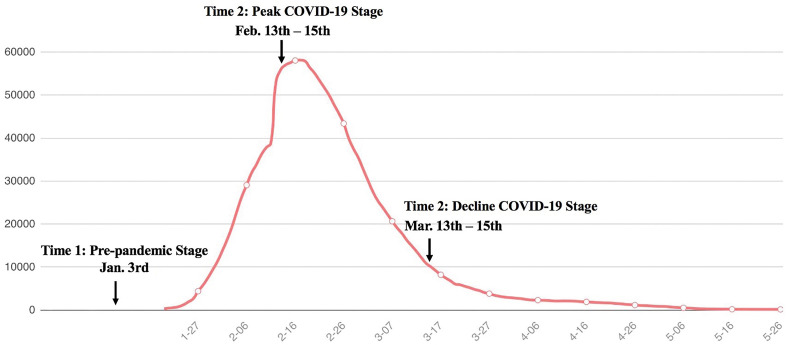
The trajectory of current confirmed cases of COVID-19 in China.

We measured the participants’ trait loneliness, perceived social support, and trait anxiety on January 3rd of 2020 when COVID-19 was not yet declared an emergent public health event. Then, we measured participants’ perceived social support and COVID-19 anxiety in mid-February (February 13th–15th; Time 2, the peak stage of the pandemic in China) and mid-March (March 13th–15th, Time 3, the decline stage of the pandemic in China; see Figure 1 for more details).

Two hundred and sixty-six Chinese adults took part in our survey, with 222 (83.46%) valid cases (filling all the questionnaires and passing test questions). Of the final participants, 54.50% (121 cases) were female, with ages ranging from 19 to 64 years (*M*_*age*_ at Time 1 = 31.53, *SD* = 8.17). Their monthly income ranged from “lower than 5,000 yuan” (49 cases, 22.07%) to “higher than 35,000 yuan” (5 cases, 2.25%). In addition, participants came from 26 provinces (11.71% from Western China, 9.91% from Northeastern China, 30.63% from East China, 22.07% from North China, 9.91% from Central China, and 15.77% from South China).

Of the 222 adults, 164 (73.87%) and 123 (54.41%) were followed at Time 2 and Time 3, respectively. Participants with complete vs. incomplete data significantly differed in their gender: c^2^(1) = 5.48, *p* = 0.019, and age: *t*(220) = 4.17, *p<*0.001.

### Measures

Table 1 presents the descriptive statistics about the measures.

**TABLE 1:** Descriptive statistics of study variables.

	***M***	***SD***	**1**	**2**	**3**	**4**	**5**	**6**	**7**	**8**	**9**	**10**	**11**	**12**	**13**
1. Trait loneliness Loneliness	22.12	6.48	–												
2. PSS (SO) T1	20.37	4.08	−0.19**	–											
3. PSS (FA) T1	22.12	4.31	−0.09	−0.51**	–										
4. PSS (FR) T1	21.02	4.10	−0.11	−0.73**	−0.52**	–									
5. PSS (SO) T2	21.54	3.36	−0.15	−0.60**	−0.30**	−0.47**	–								
6. PSS (FA) T2	23.16	4.26	−0.09	−0.41**	−0.70**	−0.38**	−0.41**	–							
7. PSS (FR) T2	22.09	2.97	−0.15	−0.58**	−0.51**	−0.66**	−0.65**	−0.55**	–						
8. PSS (SO) T3	21.17	3.70	−0.19*	−0.66**	−0.47**	−0.60**	−0.70**	−0.52**	−0.62**	–					
9. PSS (FA) T3	23.77	3.53	−0.21*	−0.34**	−0.59**	−0.32**	−0.42**	−0.70**	−0.46**	−0.59**	–				
10. PSS (FR) T3	21.73	3.23	−0.16	−0.42**	−0.40**	−0.54**	−0.49**	−0.55**	−0.67**	−0.67**	−0.56**	–			
11. CA T1	39.76	9.57	−0.19**	−0.40**	−0.45**	−0.35**	−0.31**	−0.45**	−0.33**	−0.36**	−0.40**	−0.40**	–		
12. COVA T2	24.79	6.42	−0.03	−0.03	−0.12	−0.01	−0.01	−0.01	−0.06	−0.07	−0.09	−0.09	−0.20*	–	
13. COVA T3	23.14	6.19	−0.07	−0.03	−0.12	−0.04	−0.14	−0.15	−0.08	−0.20*	−0.24**	−0.28**	−0.30**	−0.75**	–
**Covariates**															
Gender	45.53^*a*^	–	−0.09	−0.03	−0.08	−0.03	−0.08	−0.03	−0.10	−0.04	−0.02	−0.01	−0.05	−0.01	−0.02
Age	31.53	8.17	−0.18**	−0.08	−0.13	−0.04	−0.09	−0.07	−0.03	−0.12	−0.07	−0.02	−0.15*	−0.04	−0.03
Monthly Income	43.69^*b*^	–	−0.11	−0.13	−0.16*	−0.14*	−0.00	−0.20**	−0.12	−0.08	−0.02	−0.12	−0.27**	−0.07	−0.03

T1–3, Time points of assessment (T1: pre-pandemic stage; T2: peak COVID-19 stage; T3: decline COVID-19 stage); PSS (SO), Perceived Social Support (Significant Other); PSS (FA), Perceived Social Support (Family); PSS (FR), Perceived Social Support (Friends); CA, Chronic Anxiety; COVA, COVID-19 Anxiety. Means (M), standard deviations (SD). ^a^The percentage of male participants. ^*b*^The percentage of participants with monthly income between 5,000 and 10,000. *p < 0.05, **p < 0.01.

#### Trait Loneliness at Time 1 (Pre-pandemic Stage)

Trait loneliness was assessed using the 8-item loneliness subscale of the Solitude Behavior Scale (Chen et al., 2012), a valid scale for use in Chinese samples. Participants rated their levels of loneliness (e.g., “*I often feel lonely when I am alone.*”) on a 5-point scale from 1 = “*strongly disagree*” to 5 = “*strongly agree.*” We summed all items, with higher scores indicating higher levels of trait loneliness. The Cronbach’s alpha value was 0.85 in the present study.

#### Perceived Social Support at Time 1 (Pre-pandemic Stage) to 3 (Decline COVID-19 Stage)

Perceived social support was assessed using the 12-item Chinese version of the Multidimensional Scale of Perceived Social Support (MSPSS; Zimet et al., 1988). The scale included three subscales: Family support (four items; “*I get the emotional help and support I need from my family.*”), Friends support (four items; “*I have friends with whom I can share my joys and sorrows.*”), and Significant Other support (four items; “*I have a special person who is a real source of comfort to me.*”). We summed items in each subscale, with higher scores indicating higher levels of perceived social support. This scale has shown good reliability and validity in Chinese adults (Wang et al., 2017). The Cronbach’s alphas of each subscale were 0.81, 0.87, and 0.84 at Time 1, 0.84, 0.90, and 0.76 at Time 2, and 0.83, 0.81, and 0.81 at Time 3, respectively. The Cronbach’s alphas of the overall scale were 0.90 at Time 1, 0.87 at Time 2, and.86 at Time 3.

#### Chronic Anxiety at Time 1 (Pre-pandemic Stage)

Chronic anxiety was assessed via the 20-item Trait Anxiety Subscale from the Chinese version of the State-Trait Anxiety Inventory (STAI; Spielberger et al., 1983; Li and Qian, 1995). Participants rated their levels of chronic anxiety (e.g., “*I feel nervous*.”) on a 4-point scale from 1 = “*not at all*” to 4 = “*very much so.*” This scale has been well validated in Chinese adults (Chen et al., 2014). Total score was computed, with higher scores indicating higher levels of chronic anxiety. The Cronbach’s alpha in this study was 0.89.

#### COVID-19 Anxiety at Time 2 (Peak COVID-19 Stage) and 3 (Decline COVID-19 Stage)

COVID-19 anxiety was assessed via the 10-item Self-check and Self-inspect Scale for COVID-19 Anxiety (Chinese Psychological Society, 2020). Participants reported their anxious mood and behaviors after the outbreak of COVID-19 in the past month (e.g., “*I worried that the pandemic would be out of control.*”) on a 5-point scale from 1 = “*almost never*” to 5 = “*almost always.*” We summed all the items, with higher scores representing higher levels of COVID-19 anxiety. The Cronbach’s alpha values were 0.87 at Time 2 and 0.86 at Time 3.

#### Covariates at Time 1 (Pre-pandemic Stage)

Participants reported their gender (1 = male, 2 = female), age, and monthly income (from 1 = “*lower than 5,000 yuan*” to 7 = “*higher than 35,000 yuan*”). The aforementioned covariates were considered in the analyses due to their significant correlations with anxiety in previous studies (e.g., Merikangas et al., 2003; Lofors et al., 2006; McLean et al., 2011).

### Data Analysis

In order to investigate the trajectories of perceived social support (measured at all three time points) and COVID-19 anxiety (measured at Time 2 and Time 3) across time, repeated measures ANOVA were conducted. Furthermore, we considered both within-subject and between-subject variability in the models because of (1) the relatively high rate of non-random missing responses in our data, (2) the potential high variability between subjects, and (3) the fact that a participant’s response at one time point might depend on his or her response at another time point which would make the data non-independent. Thus, we conducted mixed effects modeling using the nlme package in R and considered the random effect of subjects (Pinheiro et al., 2014). Then, for perceived social support measured at all three time points, the Bonferroni-corrected *post hoc* test was conducted using the multcomp package in R (Bretz et al., 2016).

To explore the possible impact of trait loneliness (measured at Time 1) and perceived social support (measured at all three time points) on both chronic anxiety (Time 1) and COVID-19 anxiety (Time 2 and Time 3), we conducted structural equation modeling (SEM) using the lavaan package in R (Rosseel, 2012). In order to examine the interaction between trait loneliness and perceived social support on anxiety, we followed the procedure suggested by Marsh et al. (2004). After being centered, three indicators of perceived social support were multiplied by trait loneliness to form three indicators of the latent interaction term. To examine whether perceived social support buffered the harmful effects of trait loneliness to anxiety at different stages of the pandemic, three models were examined, respectively. For the model of Time 1 (Model 1), trait loneliness, perceived social support, and the latent interaction term were involved in the model as the predictors of chronic anxiety. For both models of Time 2 (Model 2) and Time 3 (Model 3), trait loneliness (measured at Time 1), the concurrent perceived social support, and their interaction term were involved in the model as the predictors of COVID-19 anxiety at that time point. In Model 2 and Model 3, given that our aim was to examine the protective effect of the concurrent perceived social support at peak and decline stages of the pandemic, perceived social support at Time 1 was included as a control variable. If a significant interaction was found in the model, the simple slopes would be examined to see the specific direction of the interaction.

We would use the following fit indices to evaluate the models’ goodness of fit: χ^2^ (Chi-Square statistics) could be accepted when the *p*-value is greater than 0.05 or when the ratio of χ^2^/*df* is less than 5, CFI (the comparative fit index) with okay fit when being more than.90, RMSEA (root-mean-square error of approximation) close to or less than 0.08, and SRMR (standardized root mean squared residual) indicating good fit when it is less than 0.08 (Kenny, 2015). However, among all the indices, χ^2^ would usually be less weighted when evaluating the model due to its sensitivity to sample size (Bentler, 1990).

Due to the relatively high percentage of missing data, Little’s (1988) missing completely at random (MCAR) test was conducted by using BaylorEdPsych package in R. We involved all the variables of this study in the test, and the results indicated that the data is non-MCAR [χ^2^(22) = 56.54 *p*< 0.001], Therefore, full information maximum likelihood estimation (FIML) was used to deal with non-MCAR missing data so that all participants would be taken into account in the analyses.

## Results

**FIGURE 2:**
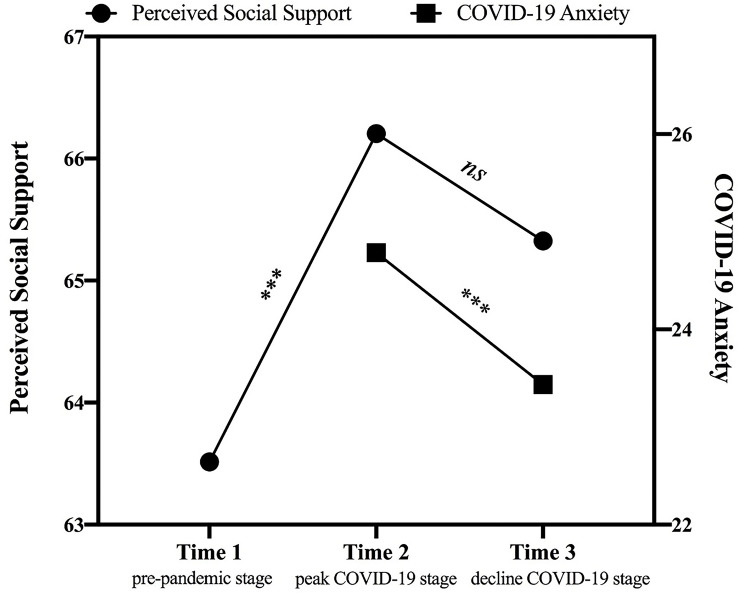
The trajectories of perceived social support (from Time 1: pre-pandemic stage to Time 3: decline COVID-19 stage) and COVID-19 anxiety (from Time 2: peak COVID-19 stage to Time 3: decline COVID-19 stage). Perceived social support was computed by summing scores of three subscales. ***p < 0.001.

First, the trajectories of perceived social support and COVID-19 anxiety were represented in Figure 2. For perceived social support, the mixed effects model had better goodness of fit as compared to the model with only fixed effects [χ^2^_*d*__*if*__*f*_(1) = 219.25, *p*< 0.001], as there was considerable variance in intercepts across participants (i.e., for different participants, there would be distinct regression expressions with different intercepts but the same slope), *SD* = 8.38, 95% CI [7.48, 9.39], accounting for 73% of the variance of the model. The results of mixed effects modeling suggested that there were discrepancies among the levels of perceived social support assessed at three time points [*F*_(2, 285)_ = 12.04, *p*< 0.001]. Specifically, perceived social support increased from Time 1 (pre-pandemic stage) to Time 2 (peak COVID-19 stage; *M*_*support*__2__–__1_ = 2.69, *p*< 0.001), and remained relatively congruent from Time 2 to Time 3 (decline COVID-19 stage; *M*_*support*__3__–__2_ = −0.88, *p* = 0.350). For COVID-19 anxiety, the mixed effects model also outperformed the model with only fixed effects [χ^2^_*d*__*if*__*f*_(1) = 99.05, *p*< 0.001], with considerable variance in intercepts across participants (*SD* = 5.49, 95% CI [4.80, 6.27]), accounting for 75% of the variance of the model. As shown in the mixed effects model, COVID-19 anxiety decreased from Time 2 (peak COVID-19 stage) to Time 3 (decline COVID-19 stage; *t* = −3.39, *p* < 0.001).

**FIGURE 3:**
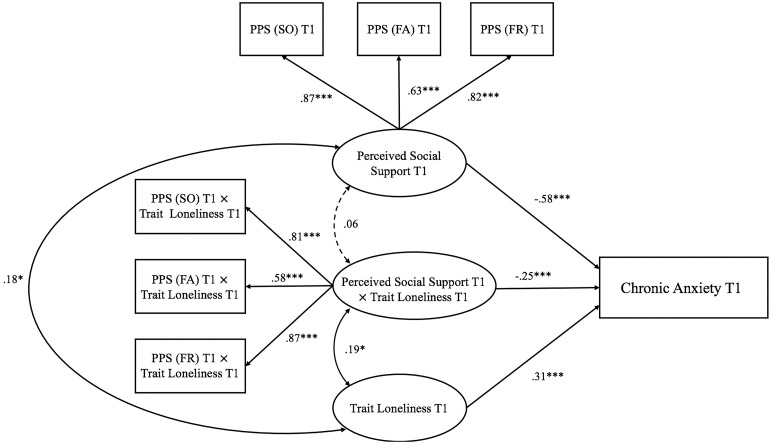
Trait loneliness interacted with perceived social support predicting chronic anxiety at Time 1 (pre-pandemic stage). Numbers represented standardized coefficients. T1–3, Time points of assessment; PSS (SO), Perceived Social Support (Significant Other); PSS (FA), Perceived Social Support (Family); PSS (FR), Perceived Social Support (Friends). *p < 0.05, ***p < 0.001.

**FIGURE 4:**
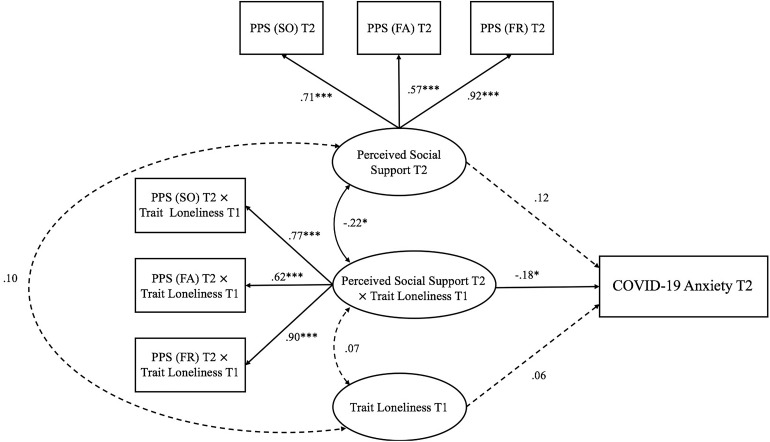
Trait loneliness interacted with perceived social support predicting COVID-19 anxiety at Time 2 (peak COVID-19 stage). Numbers represented standardized coefficients. T1–3, Time points of assessment; PSS (SO), Perceived Social Support (Significant Other); PSS (FA), Perceived Social Support (Family); PSS (FR), Perceived Social Support (Friends). Predicting pathway from perceived social support at Time 1: pre-pandemic stage to COVID-19 anxiety at Time 2: peak COVID-19 stage; β = −0.15, *p* = 0.326) was not depicted to increase clarity. *p < 0.05, ***p < 0.001.

**FIGURE 5:**
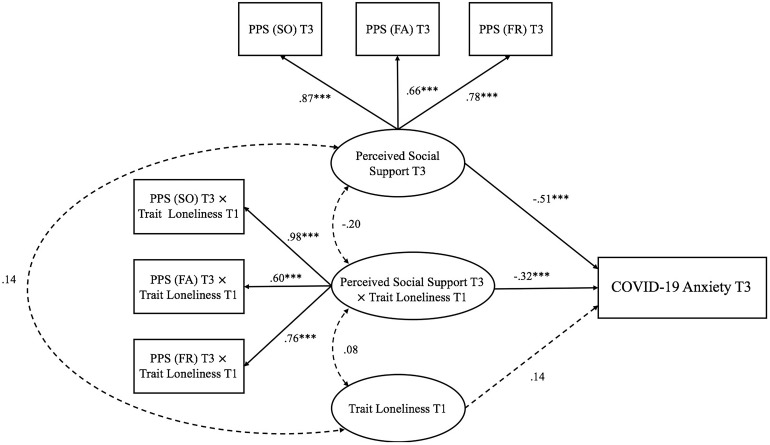
Trait loneliness interacted with perceived social support predicting COVID-19 anxiety at Time 3 (decline COVID-19 stage). Numbers represented standardized coefficients. T1–3, Time points of assessment; PSS (SO), Perceived Social Support (Significant Other); PSS (FA), Perceived Social Support (Family); PSS (FR), Perceived Social Support (Friends). Predicting pathway from perceived social support at Time 1: pre-pandemic stage to COVID-19 anxiety at Time 3: decline COVID-19 stage; β = 0.20, *p* = 0.164) was not depicted to increase clarity. ***p < 0.001.

**FIGURE 6:**
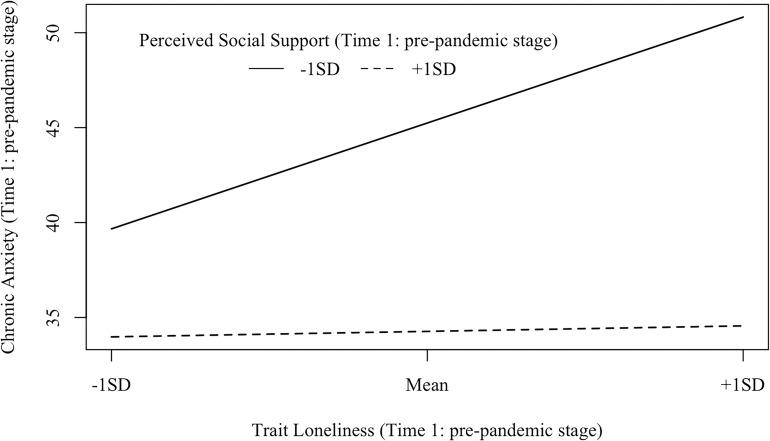
Interaction effects between trait loneliness (Time 1: pre-pandemic stage) and perceived social support (Time 1: pre-pandemic stage) on chronic anxiety (Time 1: pre-pandemic stage). β (–1 *SD*) = 0.58, *p*<0.001; β (+1 *SD*) = 0.03, *p* = 0.734.

Second, three models examining the interactive effect of trait loneliness and perceived social support on anxiety at different stages of the pandemic were represented in Figures 3–5. For Model 1 (Time 1: pre-pandemic stage; see Figure 3), the goodness of fit was generally acceptable [χ^2^(16) = 43.140, *p*< 0.001, CFI = 0.95, RMSEA = 0.087, SRMR = 0.058]. Both perceived social support (β = −0.58, *p <* 0.001) and trait loneliness (β = 0.31, *p*< 0.001) independently predicted chronic anxiety. The latent interaction term also significantly predicted chronic anxiety (β = −0.25, *p*< 0.001, D*R*^2^ = 0.058). Specifically (see Figure 6), for individuals with lower perceived social support (−1 *SD*), trait loneliness predicted heightened level of chronic anxiety (β = 0.58, *p <* 0.001). However, for those with higher levels of perceived social support (+1 *SD*), trait loneliness did not predict chronic anxiety (β = 0.03, *p* = 0.734).

**FIGURE 7:**
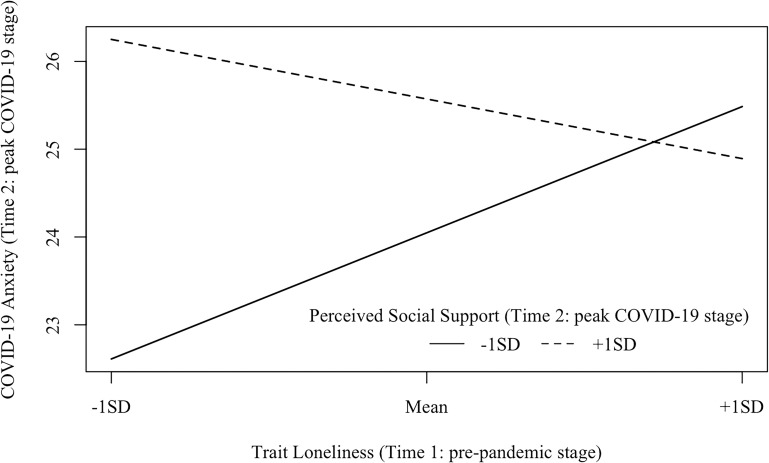
Interaction effects between trait loneliness (Time 1: pre-pandemic stage) and perceived social support (Time 2: peak COVID-19 stage) on COVID-19 anxiety (Time 2: peak COVID-19 stage). β (–1 *SD*) = 0.22, *p* = 0.067; β (+1 *SD*) = −0.11, *p* = 0.335.

Model 2 (Time 2: peak COVID-19 stage; see Figure 4) examined the interaction between trait loneliness (measured at Time 1) and perceived social support (measured at Time 2) on COVID-19 anxiety at the peak stage of COVID-19 in China (i.e., Time 2) after controlling for baseline perceived social support assessed at the pre-pandemic stage (i.e., Time 1). The model fit the data well [χ^2^(33) = 52.26, *p* = 0.018, CFI = 0.98, RMSEA = 0.051, SRMR = 0.041]. Neither trait loneliness (β = 0.06, *p* = 0.477) nor the concurrently perceived social support (β = 0.12, *p* = 0.413) predicted COVID-19 anxiety. However, there was a significant interaction between trait loneliness and perceived social support on COVID-19 anxiety (β = −0.18, *p* = 0.042, D*R*^2^ = 0.029). Specifically (see Figure 7), for individuals with low (−1 *SD*) perceived social support, trait loneliness marginally, but positively, predicted COVID-19 anxiety (β = 0.22, *p* = 0.067); for individuals with high (+1 *SD*) perceived social support, trait loneliness did not predict COVID-19 anxiety (β = −0.11, *p* = 0.335).

**FIGURE 8:**
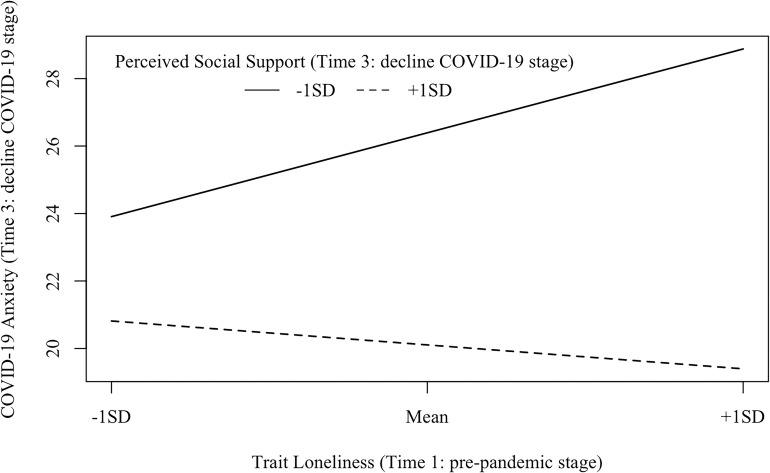
Interaction effects between trait loneliness (Time 1: pre-pandemic stage) and perceived social support (Time 3: decline COVID-19 stage) on COVID-19 anxiety (Time 3: decline COVID-19 stage). β (–1 *SD*) = 0.40, *p* = 0.001; β (+1 *SD*) = −0.11, *p* = 0.40.

Model 3 (Time 3: decline COVID-19 stage; see Figure 5) examined the interaction between trait loneliness (measured at Time 1) and perceived social support (measured at Time 3) on COVID-19 anxiety at the decline stage of COVID-19 in China (i.e., Time 3). The goodness of fit was acceptable [χ^2^(33) = 63.18, *p* = 0.001, CFI = 0.96, RMSEA = 0.064, SRMR = 0.065]. COVID-19 anxiety at Time 3 could be directly predicted by perceived social support (β = −0.51, *p*< 0.001), but not trait loneliness (β = 0.14, *p* = 0.107). The latent interaction term significantly predicted COVID-19 anxiety (β = −0.32, *p*< 0.001, D*R*^2^ = 0.095). Specifically (see Figure 8), trait loneliness positively predicted COVID-19 anxiety for individuals with a low level of perceived social support (−1 *SD*, β = 0.40, *p* = 0.001), but not for those with a high level of perceived social support (+1 *SD*, β = −0.11, *p* = 0.40).

Finally, considering the potential impact of covariates, we then examined the model again controlling for age, gender, and monthly income for all three models. In addition, chronic anxiety measured at Time 1 was also controlled in Model 2 and Model 3. For Model 1 and Model 3, the results stayed the same after considering the covariates. However, for Model 2, after entering the covariates into the model, the interaction between perceived social support and trait loneliness was no longer significant (β = −0.12, *p* = 0.177). In order to investigate what the exact factor leading to the insignificance of the interaction in Model 2 was, we tested the model by controlling only one variable at a time. The results suggested that both monthly income (β = −0.13, *p* = 0.123) and chronic anxiety at Time 1 (β = −0.17, *p* = 0.050) accounted for the insignificance of the interaction between trait loneliness and perceived social support in the model.

## Discussion

To examine the relationship among trait loneliness, perceived social support, and anxiety among Chinese adults, we conducted a three-wave longitudinal study during three stages of the COVID-19 pandemic. There were three main findings in the present study. First, perceived social support moderated the relationship between loneliness and chronic anxiety. Second, perceived social support sharply increased from the pre-pandemic stage to the peak COVID-19 stage, and remained relatively stable from the peak to the decline COVID-19 stage; COVID-19 anxiety decreased from the peak to the decline COVID-19 stage. Third, perceived social support moderated the relationship between loneliness and COVID-19 anxiety at the peak and decline COVID-19 stages of the pandemic. We discuss these in more detail below.

First, the fluctuation of perceived social support with the development of the COVID-19 pandemic might be explained by the terror management theory (Greenberg et al., 1994). The outbreak of COVID-19 makes mortality salient, which may then arouse people’s anxiety. To resist death anxiety, people might engage in social interaction and acquire social support (Heine et al., 2006; Pinson, 2010).

Second, the finding that perceived social support moderated the relationship between loneliness and anxiety across three stages of the COVID-19 pandemic was consistent with the social support buffering hypothesis (Cohen and Pressman, 2004). At the pre-pandemic stage, lonely people’s maladaptive social attention, cognition, and emotion may contribute to high chronic anxiety in daily life (the pre-pandemic stage; Hawkley and Cacioppo, 2010). In this case, heightened levels of perceived social support might provide them with a sense of companionship and belongingness, which can serve as a psychological resource to mitigate chronic anxiety (Taylor and Broffman, 2011). Furthermore, the moderating effect of social support in the prediction of loneliness to COVID-19 anxiety remained consistent across the peak and decline stages of the COVID-19 pandemic. As an acute stressor, the outbreak of the COVID-19 pandemic and its related social isolation policy might intensify lonely people’s vulnerability to anxiety (Brooks et al., 2020). However, the perception of more social support can make them feel cared for, understood, and valued by others, which can strengthen one’s self-efficacy in coping with the uncertainty of the future (Casale and Flett, 2020). Confirming and extending previous findings on the benefits of social support in disasters (Arnberg et al., 2012; Gabert-Quillen et al., 2012; Guilaran et al., 2018; Skalski et al., 2020), findings from the present study demonstrated the protective role of social support for lonely people across different stages of the COVID-19 pandemic.

Third, when controlling for the covariates (e.g., monthly income and chronic anxiety at the pre-pandemic stage), the moderating effect of perceived social support in the relationship between loneliness and COVID-19 anxiety at the peak COVID-19 stage was insignificant. One potential reason for this finding is that there are many other factors apart from loneliness and social support that might give rise to people’s anxiety at the peak COVID-19 stage, such as potential financial difficulties, relationship breakdowns, and uncertainty about the future (Fiorillo and Gorwood, 2020; Holmes et al., 2020). These factors might weaken the predictive power of the interaction of loneliness and social support to COVID-19 anxiety.

Moreover, we also tentatively analyzed whether different social support domains (i.e., significant other, family, and friends) played different roles in the relationship between loneliness and anxiety across the three stages of the pandemic (see Supplementary Table S1). The results showed that at the pre-pandemic and decline COVID-19 stages, perceived social support from significant others and friends buffered the detrimental effects of trait loneliness to chronic anxiety and COVID-19 anxiety. However, the results inversed at the peak COVID-19 stage, such that only perceived social support from family buffered the detrimental effects from trait loneliness to COVID-19 anxiety, whereas perceived social support from significant others and friends did not moderate the relationship between trait loneliness and COVID-19 anxiety. Therefore, it seems that in daily life and at times when the acute stressor has generally passed, social support from significant others and friends tends to protect lonely people from anxiety, whereas this source of social support was weakened when confronting the life-threatening stressor and shelter-in-place policy. Instead, the connection among family members had become the most important relationship for most people at the peak COVID-19 stage when almost everyone was confined at home. Future studies are needed to further examine the role of the family system (e.g., parent–child relationship, marital relationship, and family environment) in protecting individuals from anxiety.

Findings from the present study point to some practical implications for public policies and intervention strategies. First, although being isolated, people could strengthen their emotional connection with others through network-based ways, which might lower their COVID-19 anxiety. Second, mental health organizations and practitioners should consider developing online social support programs to cater to the public’s need for more social connections. Third, policymakers are encouraged to find ways to address individuals’ financial stress and threat of unemployment. Finally, we should pay more attention to lonely individuals, who are sensitive to both chronic and state stressors. For example, clinicians can use crisis intervention programs together with cognitive behavioral therapy to correct the irrational beliefs and negative thoughts of lonely individuals about the crisis (Diefenbach and Goethe, 2006; Subramanyam et al., 2018).

Some limitations of the current study, as well as future directions, are worth noting. First, all measurements were self-reported in our study, which might inflate the correlations among variables. Future studies are suggested to use multiple approaches (e.g., daily diary) and multi-informant methods. Second, most of the participants in the sample are middle-aged. However, recent reviews and studies have shown that loneliness and social isolation might have a more negative influence on children and the elderly (Armitage and Nellums, 2020; Brooke and Jackson, 2020; Xiang et al., 2020). Therefore, studies focusing on protective factors for the mental health of children and the elderly under the social distancing policy are warranted. Third, social support can be further divided into several domains (e.g., instrumental support and emotional support; House, 1981). Cutrona and Russell (1990) proposed that emotional support could serve as a stronger buffer against the harmful influence of the uncontrollable stressors than other types of support. Future studies are needed to examine the protective role of emotional support for the public’s health in the COVID-19 period. Fourth, the current study only had information on individuals’ income before the outbreak of the pandemic, without considering the updated income which might be influenced by the pandemic situation (e.g., losing jobs and receiving no income). It would be beneficial for future studies to explore the effects of updated income on the relationship between loneliness and COVID-19 anxiety.

## Data Availability Statement

The raw data supporting the conclusions of this article will be made available by the authors, without undue reservation.

## Ethics Statement

The studies involving human participants were reviewed and approved by the ethics board of the Faculty of Psychology, Beijing Normal University. The patients/participants provided their written informed consent to participate in this study.

## Author Contributions

JX and YW designed the study. JX, JO, SL, ZW, EC, CN, and YW wrote and critically revised the manuscript. All authors reviewed, edited and approved the manuscript.

## Conflict of Interest

The authors declare that the research was conducted in the absence of any commercial or financial relationships that could be construed as a potential conflict of interest.

## Footnotes

1 www.wjx.cn

## References

[B1] Antonovsky A. (1987). *Unraveling the Mystery of Health–How People Manage Stress and Stay Well.* San Francisco: Jossey-Bass Publishers.

[B2] Armitage R. Nellums L. B. (2020). COVID-19 and the consequences of isolating the elderly. *Lancet Public Health* 5:e256 10.1016/s2468-2667(20)30061-x PMC7104160 32199471

[B3] Arnberg F. K. Hultman C. M. Michel P. O. Lundin T. (2012). Social support moderates posttraumatic stress and general distress after disaster. *J. Trauma. Stress* 25 721–727. 10.1002/jts.21758 23184348

[B4] Banerjee D. Rai M. (2020). Social isolation in Covid-19: the impact of loneliness. *Int. J. Soc. Psychiatry* 66 525–527. 10.1177/0020764020922269 32349580 PMC7405628

[B5] Bao Y. Sun Y. Meng S. Shi J. Lu L. (2020). 2019-nCoV epidemic: address mental health care to empower society. *Lancet* 395 e37–e38. 10.1016/s0140-6736(20)30309-3 32043982 PMC7133594

[B6] Bentler P. M. (1990). Comparative fit indexes in structural models. *Psychol. Bull.* 107:238. 10.1037/0033-2909.107.2.238 2320703

[B7] Bretz F. Hothorn T. Westfall P. (2016). *Multiple Comparisons Using R.* Boca Raton, FL: CRC Press.

[B8] Brooke J. Jackson D. (2020). Older people and COVID−19: isolation, risk and ageism. *J. Clin. Nurs.* 29 2044–2046. 10.1111/jocn.15274 32239784

[B9] Brooks S. K. Webster R. K. Smith L. E. Woodland L. Wessely S. Greenberg N. (2020). The psychological impact of quarantine and how to reduce it: rapid review of the evidence. *Lancet* 395 912–920. 10.2139/ssrn.3532534 32112714 PMC7158942

[B10] Bureau of Disease Prevention and Control (2020). http://www. nhc.gov.cn/jkj/s3577/202001/e5e8c983baba4c1589512e6c99fdaa4e.shtml (accessed May 24, 2020).

[B11] Casale S. Flett G. L. (2020). Interpersonally-based fears during the COVID-19 pandemic: reflections on the fear of missing out and the fear of not mattering constructs. *Clin. Neuropsychiatry* 17 88–93. 10.36131/CN20200211 PMC8629079 34908975

[B12] Chen X. L. Dai X. Y. Bao L. Wang M. Liu M. (2012). Development and psychometric properties of the solitude behavior scale. *Chinese J. Clin. Psychol.* 20 1–4. 10.16128/j.cnki.1005-3611.2012.01.001

[B13] Chen X. L. Song Y. P. Sun H. W. (2014). The relationship between general self-efficacy and state-trait anxiety of adolescents. *China J. Health Psychol.* 22 418–419. 10.13342/j.cnki.cjhp.2014.03.044

[B14] Chinese Psychological Society (2020). *Self-check and Self-inspect Scale for COVID-19 Anxiety.* on-line, welcome to scan and fill in the answer. Available online at: https://mp.weixin.qq.com/s/rtlu22EAwkEHd2PKg-pXmg (accessed April 9, 2020).

[B15] Cohen S. Pressman S. (2004). “Stress-buffering hypothesis,” in *Encyclopedia of Health and Behavior*, ed. Anderson N. B. (Thousand Oaks, CA: Sage), 696–697. 10.4135/9781412952576.n200

[B16] Cutrona C. E. Russell D. W. (1990). “Type of social support and specific stress: toward a theory of optimal matching,” in *Social Support: An Interactional View*, eds Sarason B. R. Sarason I G. Pierce G. R. (Hoboken, NJ: John Wiley and Sons), 319–366.

[B17] Diefenbach G. J. Goethe J. (2006). Clinical interventions for late-life anxious depression. *Clin. Interv. Aging* 1:41. 10.2147/ciia.2006.1.1.41 18047256 PMC2682453

[B18] Fiorillo A. Gorwood P. (2020). The consequences of the COVID-19 pandemic on mental health and implications for clinical practice. *Eur. Psychiatry* 63 1–2. 10.1192/j.eurpsy.2020.35 32234102 PMC7156565

[B19] Ford T. Vizard T. Sadler K. McManus S. Goodman A. Merad S. (2020). Data resource profile: the mental health of children and young people surveys (MHCYP). *Int. J. Epidemiol.* 49:259. 10.1093/ije/dyz259 31953946

[B20] Gabert-Quillen C. A. Irish L. A. Sledjeski E. Fallon W. Spoonster E. Delahanty D. L. (2012). The impact of social support on the relationship between trauma history and posttraumatic stress disorder symptoms in motor vehicle accident victims. *Int. J. Stress Manag.* 19 69–79. 10.1037/a0026488 22468117 PMC3314329

[B21] Galea S. Merchant R. M. Lurie N. (2020). The mental health consequences of COVID-19 and physical distancing: The need for prevention and early intervention. *JAMA Intern. Med.* 180 817–818. 10.1001/jamainternmed.2020.1562 32275292

[B22] Greenberg J. Pyszczynski T. Solomon S. Simon L. Breus M. (1994). Role of consciousness and accessibility of death-related thoughts in mortality salience effects. *J. Pers. Soc. Psychol.* 67 627–637. 10.1037/0022-3514.67.4.627 7965609

[B23] Greenstone M. Nigam V. (2020). *Does social distancing matter? University of Chicago, Becker Friedman Institute for Economics Working Paper No. 2020-26.* Chicago: University of Chicago, 10.2139/ssrn.3561244

[B24] Guilaran J. de Terte I. Kaniasty K. Stephens C. (2018). Psychological outcomes in disaster responders: A systematic review and meta-analysis on the effect of social support. *Int. J. Disaster Risk Sci.* 9 344–358. 10.1007/s13753-018-0184-7

[B25] Guo L. Xu P. R. Yao F. Zhang F. Y. Qi L. Yang F. H. (2020). The effect of acute stress disorder on Chinese public’s negative affective conditions under the severe pandemic: the moderating role of social support. *J. Southwest U.* 42 1–10.

[B26] Hawkley L. C. Cacioppo J. T. (2010). Loneliness matters: A theoretical and empirical review of consequences and mechanisms. *Ann. Behav. Med.* 40 218–227. 10.1007/s12160-010-9210-8 20652462 PMC3874845

[B27] Heine S. J. Proulx T. Vohs K. D. (2006). The meaning maintenance model: On the coherence of social motivations. *Pers. Soc. Psychol. Rev.* 10 88–110. 10.1207/s15327957pspr1002_1 16768649

[B28] Holmes E. A. O’Connor R. C. Perry V. H. Tracey I. Wessely S. Arseneault L. (2020). Multidisciplinary research priorities for the COVID-19 pandemic: a call for action for mental health science. *Lancet Psychiatry* 7 547–560. 10.1016/S2215-0366(20)30168-1 32304649 PMC7159850

[B29] House J. S. (1981). *Work Stress and Social Support.* Reading, MA: Addison Wesley.

[B30] Huang Y. Zhao N. (2020). Generalized anxiety disorder, depressive symptoms and sleep quality during COVID-19 outbreak in China: a web-based cross-sectional survey. *Psychiatry Res.* 288:112954 10.21203/rs.3.rs-17172/v1 PMC7152913 32325383

[B31] Kenny D. A. (2015). *Measuring Model Fit.* Available online at: http://davidakenny.net/cm/fit.htm (accessed May 20, 2020).

[B32] Li W. L. Qian M. Y. (1995). Revision of Chinese college students’ norm of state trait anxiety scale. *Acta Sci. Nat. Univ. Pekin.* 31 108–112. 10.13209/j.0479-8023.1995.014

[B33] Little R. J. (1988). A test of missing completely at random for multivariate data with missing values. *J. Am. Stat. Assoc.* 83 1198–1202. 10.2307/2290157

[B34] Lofors J. Ramírez-León V. Sundquist K. (2006). Neighbourhood income and anxiety: a study based on random samples of the Swedish population. *Eur. J. Public Health* 16 633–639. 10.1093/eurpub/ckl026 16641161

[B35] Marsh H. W. Wen Z. Hau K. T. (2004). Structural equation models of latent interactions: evaluation of alternative estimation strategies and indicator construction. *Psychol. Methods* 9 275–300. 10.1037/1082-989X.9.3.275 15355150

[B36] Masten A. S. (2001). Ordinary magic: resilience processes in development. *Am. Psychol.* 56:227. 10.1037/0003-066x.56.3.227 11315249

[B37] McLean C. P. Asnaani A. Litz B. T. Hofmann S. G. (2011). Gender differences in anxiety disorders: prevalence, course of illness, comorbidity and burden of illness. *J. Psychiatry Res.* 45 1027–1035. 10.1016/j.jpsychires.2011.03.006 21439576 PMC3135672

[B38] Merikangas K. R. Zhang H. Avenevoli S. Acharyya S. Neuenschwander M. Angst J. (2003). Longitudinal trajectories of depression and anxiety in a prospective community study: the Zurich cohort study. *Arch. Gen. Psychiatry* 60 993–1000. 10.1001/archpsyc.60.10.993 14557144

[B39] Oh H. J. Ozkaya E. LaRose R. (2014). How does online social networking enhance life satisfaction? The relationships among online supportive interaction, affect, perceived social support, sense of community, and life satisfaction. *Comput. Hum. Behav.* 30 69–78. 10.1016/j.chb.2013.07.053

[B40] Othman N. (2020). Depression, anxiety, and stress in the time of COVID-19 pandemic in Kurdistan region. *Iraq. Kurdistan J. Appl. Res.* 5 37–44. 10.24017/covid.5

[B41] Pancani L. Marinucci M. Aureli N. Riva P. (2020). *Forced Social Isolation and Mental Health: A Study on 1006 Italians Under COVID-19 Lockdown.* Available online at: https://psyarxiv.com/uacfj/ (accessed May 05, 2020). 10.3389/fpsyg.2021.663799 PMC8175900 34093358

[B42] Peplau L. A. Perlman D. (1982). “Perspectives on loneliness,” in *Loneliness: A Sourcebook of Current Theory, Research and Therapy*, eds Peplau L. A. Perlman D. (New York, NY: Wiley), 1–18.

[B43] Pinheiro J. Bates D. DebRoy S. Sarkar D. R Core Team. (2014). *nlme: Linear and nonlinear mixed effects models (R package version 3.1-128).* Available online at: http://CRAN.R-project.org/ package=nlme

[B44] Pinson M. W. (2010). *Effect of Loneliness on Older Adults’ Death Anxiety.* Doctoral dissertation North Texas: University of North Texas.

[B45] Rogers J. P. Chesney E. Oliver D. Pollak T. A. McGuire P. Fusar-Poli P. (2020). Psychiatric and neuropsychiatric presentations associated with severe coronavirus infections: a systematic review and meta-analysis with comparison to the COVID-19 pandemic. *Lancet Psychiatry* 7 611–627. 10.1016/S2215-0366(20)30203-0 32437679 PMC7234781

[B46] Rosseel Y. (2012). Lavaan: An R package for structural equation modeling and more. Version 0.5–12 (BETA). *J. Stat. Soft.* 48 1–36. 10.18637/jss.v048.i02

[B47] Roy D. Tripathy S. Kar S. K. Sharma N. Verma S. K. Kaushal V. (2020). Study of knowledge, attitude, anxiety and perceived mental healthcare need in Indian population during COVID-19 pandemic. *Asian J. Psychiatry* 51:102083. 10.1016/j.ajp.2020.102083 32283510 PMC7139237

[B48] Shevlin M. McBride O. Murphy J. Miller J. G. Hartman T. K. Levita L. (2020). Anxiety, depression, traumatic stress, and COVID-19 related anxiety in the UK general population during the COVID-19 pandemic. *BJPsych Open* 6:e125. 10.1192/bjo.2020.109 PMC7573460 33070797

[B49] Skalski S. Uram P. Dobrakowski P. Kwiatkowska A. (2020). *The link between ego-resiliency, social support, SARS-CoV-2 anxiety and trauma effects. Polish adaptation of the Coronavirus Anxiety Scale.* Available online at: https://psyarxiv.com/28tnw/ (accessed May 18, 2020). 10.1016/j.paid.2020.110540 PMC7670928 33223590

[B50] Spielberger C. D. Gonzalez-Reigosa F. Martinez-Urrutia A. Natalicio L. Natalicio D. S. (1971). Development of the Spanish edition of the state-trait anxiety inventory. *Interamerican J. Psychol.* 5 145–158. 10.1002/9780470479216.corpsy0943

[B51] Spielberger C. D. Gorsuch R. L. Lushene R. Vagg P. R. Jacobs G. A. (1983). *Manual for the State-Trait Anxiety Inventory (Form Y).* Palo Alto, CA: Consulting Psychologists Press.

[B52] Spithoven A. W. Bijttebier P. Goossens L. (2017). It is all in their mind: a review on information processing bias in lonely individuals. *Clin. Psychol. Rev.* 58 97–114. 10.1016/j.cpr.2017.10.003 29102150

[B53] Subramanyam A. A. Kedare J. Singh O. Pinto C. (2018). Clinical practice guidelines for geriatric anxiety disorders. *Indian J. Psychiatry* 60 S371–S382. 10.4103/0019-5545.224476 29535471 PMC5840911

[B54] Taylor S. E. Broffman J. I. (2011). Psychosocial resources: functions, origins, and links to mental and physical health. *Adv. Exp. Soc. Psychol.* 44 1–57. 10.1016/B978-0-12-385522-0.00001-9

[B55] Wang Y. Wan Q. Huang Z. Huang L. Kong F. (2017). Psychometric Properties of multi-dimensional scale of perceived social support in Chinese parents of children with cerebral palsy. *Front. Psychol.* 8:2020. 10.3389/fpsyg.2017.02020 29209254 PMC5702313

[B56] World Health Organization (2020a). *Coronavirus disease (COVID-19) advice for the public.* https://www.who.int/emergencies/diseases/novel-coronavirus-2019/advice-for-public (accessed May 27, 2020).

[B57] World Health Organization (2020b). *WHO announces COVID-19 outbreak a pandemic.* http://www.euro.who.int/en/health-topics/health-emergencies/coronavirus-covid-19/news/news/2020/3/who-announces-covid-19-outbreak-a-pandemic (accessed May 23, 2020).

[B58] World Health Organization (2020c). *WHO Coronavirus Disease (COVID-19) Dashboard.* https://covid19.who.int/?gclid=EAIaIQobChMIntrH6YLK 6QIVQdeWCh0VyApcEAAYASABEgLrXvD_BwE (accessed May 28, 2020).

[B59] Xiang M. Zhang Z. Kuwahara K. (2020). Impact of COVID-19 pandemic on children and adolescents’ lifestyle behavior larger than expected. *Prog. Cardiovasc. Dis.* 63 531–532. 10.1016/j.pcad.2020.04.013 32360513 PMC7190470

[B60] Zimet G. D. Dahlem N. W. Zimet S. G. Farley G. K. (1988). The multidimensional scale of perceived social support. *J. Pers. Assess.* 52 30–41. 10.1207/s15327752jpa5201_2 2280326

