# Supplementary material for: Perceived Social Support Protects Lonely People Against COVID-19 Anxiety: A Three-Wave Longitudinal Study in China
Source: Front Psychol. 2020 Nov 6;11:566965. doi: 10.3389/fpsyg.2020.566965 (PMC7677578; doi:10.3389/fpsyg.2020.566965)
Supplement: Supplementary file 1 [file Data_Sheet_1.pdf]

Supplementary Material

1 Interaction between Domains of Perceived Social Support and Trait Loneliness

The interactions between trait loneliness (measured at Time 1: pre-pandemic stage) and perceived social support from significant other, family, and friends (measured at Time 1: pre-pandemic stage) were examined respectively. For the interaction between trait loneliness and perceived social support from significant other, both perceived social support from significant other ($\beta = -.30$, $p < .001$) and trait loneliness ($\beta = .25$, $p < .001$) independently predicted chronic anxiety. The interaction of these two could also significantly predict chronic anxiety ($\beta = -.19$, $p < .001$, $\Delta R^2 = .034$). Specifically, for individuals with lower perceived social support from significant other (-1 *SD*), trait loneliness predicted heightened level of chronic anxiety ($\beta = .45$, $p < .001$). However, for those with higher level of perceived social support from significant other (+1 *SD*), trait loneliness did not predict chronic anxiety ($\beta = .06$, $p = .446$). For the interaction between trait loneliness and perceived social support from family, both perceived social support from family ($\beta = -.34$, $p < .001$) and trait loneliness ($\beta = .26$, $p < .001$) independently predicted chronic anxiety, but there is no interaction between these two variables ($\beta = -.11$, $p = .052$, $\Delta R^2 = .012$). For the interaction between trait loneliness and perceived social support from friends, perceived social support from friends could not independently predict chronic anxiety ($\beta = -.00$, $p = .962$), but trait loneliness could ($\beta = .27$, $p < .001$). The interaction of these two could also significantly predict chronic anxiety ($\beta = -.22$, $p < .001$, $\Delta R^2 = .038$). Specifically, for individuals with lower perceived social support from friends (-1 *SD*), trait loneliness predicted heightened level of chronic anxiety ($\beta = .48$, $p < .001$). However, for those with higher level of perceived social support from friends (+1 *SD*), trait loneliness did not predict chronic anxiety ($\beta = .05$, $p = .522$).

The interactions between trait loneliness (measured at Time 1: pre-pandemic stage) and perceived social support from significant other, family, and friends (measured at Time 2: peak COVID-19 stage) were examined respectively. For the interaction between trait loneliness and perceived social support from significant other, both perceived social support from significant other ($\beta = -.16$, $p = .184$) and trait loneliness ($\beta = .05$, $p = .557$) could not independently predict COVID-19 anxiety, and there was no interaction between these two variables when predicting COVID-19 anxiety ($\beta = -.11$, $p = .182$, $\Delta R^2 = .011$). For the interaction between trait loneliness and perceived social support from family, both perceived social support from family ($\beta = .08$, $p = .526$) and trait loneliness ($\beta = .06$, $p = .508$) could not independently predict COVID-19 anxiety, but the interaction of these two could significantly predict COVID-19 anxiety ($\beta = -.18$, $p = .044$, $\Delta R^2 = .025$). Specifically, for individuals with lower perceived social support from family (-1 *SD*), trait loneliness marginally and positively predicted the level of COVID-19 anxiety ($\beta = .23$, $p = .062$). However, for those with higher level of perceived social support from family (+1 *SD*), trait loneliness did not predict COVID-19 anxiety ($\beta = -.12$, $p = .319$). For the interaction between trait loneliness and perceived social support from friends, both perceived social support from friend ($\beta = .18$, $p = .170$) and trait loneliness ($\beta = .07$, $p = .448$) could not independently predict COVID-19 anxiety, and there was no interaction between these two variables when predicting COVID-19 anxiety ($\beta = -.14$, $p = .099$, $\Delta R^2 = .017$).

The interactions between trait loneliness (measured at Time 1: pre-pandemic stage) and perceived social support from significant other, family, and friends (measured at Time 3: decline COVID-19 stage) were examined respectively. For the interaction between trait loneliness and perceived social support form significant other, both perceived social support from significant other (β = -.10, p = .496) and trait loneliness (β = .16, p = .089) could not independently predict COVID-19 anxiety, but the interaction of these two could significantly predict COVID-19 anxiety (β = −.30, p < .001, ΔR^2 = .095). Specifically, for individuals with lower perceived social support from significant other (-1 *SD*), trait loneliness could marginally and positively predict the level of COVID-19 anxiety (β = .46, p = .001). However, for those with higher level of perceived social support from significant other (+1 *SD*), trait loneliness did not predict COVID-19 anxiety (β = -.14, p = .240). For the interaction between trait loneliness and perceived social support from family, both perceived social support from family (β = −.15, p = .282) and trait loneliness (β = .12, p = .221) could not independently predict COVID-19 anxiety, and there is no interaction between these two variables when predicting COVID-19 anxiety (β = −.11, p = .206, ΔR^2 = .013). For perceived social support from friends, it could independently predict COVID-19 anxiety (β = −.31, p = .024), but trait loneliness could not (β = .16, p = .102). The interaction of these two could significantly predict COVID-19 anxiety (β = −.21, p = .016, ΔR^2 = .045). Specifically, for individuals with lower perceived social support from friends (-1 *SD*), trait loneliness predicted heightened level of COVID-19 anxiety (β = .27, p = .009). However, for those with higher level of perceived social support from friends (+1 *SD*), trait loneliness had no impact on COVID-19 anxiety (β = -.05, p = .654).

2 Supplementary Table

2.1 Supplementary Table 1. Summary of the interacting effects between domains of perceived social support and trait loneliness on anxiety.

	β	*SE*	*p*	ΔR^2
**T1 significant other × loneliness ➔ chronic anxiety**	**-.19**	**.06**	**< .001**	**.034**
T1 family × loneliness ➔ chronic anxiety	-.11	.06	.052	.012
**T1 friends × loneliness ➔ chronic anxiety**	**-.22**	**.06**	**< .001**	**.038**
T2 significant other × loneliness ➔ COVID-19 anxiety	-.11	.08	.182	.011
**T2 family × loneliness ➔ COVID-19 anxiety**	**-.18**	**.09**	**.044**	**.025**
T2 friends × loneliness ➔ COVID-19 anxiety	-.14	.08	.099	.017
**T3 significant other × loneliness ➔ COVID-19 anxiety**	**-.30**	**.08**	**< .001**	**.095**
T3 family × loneliness ➔ COVID-19 anxiety	-.11	.09	.206	.013
**T3 friends × loneliness ➔ COVID-19 anxiety**	**-.21**	**.09**	**.016**	**.045**

Notes. T1 – T3 = Time points of assessment (T1: pre-pandemic stage; T2: peak COVID-19 stage; T3: decline COVID-19 stage). significant other = perceived social support from significant other; family = perceived social support from family; friends = perceived social support from friends. Gender, age, and monthly income were entered into the regression model as covariates. The main effects of perceived social support from different domains and trait loneliness had been entered into the regression models before entering the interaction terms.
